# Knowledge, Perception and Utilization of Postnatal Care of Mothers in Gondar Zuria District, Ethiopia: A Cross-Sectional Study

**DOI:** 10.1007/s10995-014-1474-3

**Published:** 2014-04-26

**Authors:** Fikirte Tesfahun, Walelegn Worku, Fekadu Mazengiya, Manay Kifle

**Affiliations:** 1 Yekokeb Berhan Program for Highly Vulnerable Children, Mahibere Hiwot Reproductive Health and Social Development Organazation, P. O. Box. No. 631, Gondar, Ethiopia; 2 Department of Environmental and Occupational Health and Safety, College of Medicine and Health Science, University of Gondar, P. O. Box. No. 196, Gondar, Ethiopia; 3 Department of Midwifery, College of Medicine and Health Science, University of Gondar, P. O. Box. No. 196, Gondar, Ethiopia

**Keywords:** Knowledge of mothers, Mothers’ perception, Utilization of postnatal care, Lack of service, Ethiopia

## Abstract

Mothers and their newborns are vulnerable to illnesses and deaths during the postnatal period. More than half a million women each year die of causes related to pregnancy and childbirth. The majority of deaths occur in less developed countries. Utilization of postnatal care (PNC) service in Ethiopia is low due to various factors. These problems problem significantly hold back the goal of decreasing maternal and child mortality. To assess mothers’ knowledge, perception and utilization of PNC in the Gondar Zuria District, Ethiopia. Our study is a community-based, cross-sectional study supported by a qualitative study conducted among 15–49 years mothers who gave birth during the last year. A multistage sampling technique was used to selected participants; structured questionnaires and focus group discussions were used to collect data. Data were entered into EPI info version 3.5.1 and exported into SPSS version 16.0 for the quantitative study and thematic framework analysis was applied to the qualitative portion. The majority of the women (84.39 %) were aware and considered PNC necessary (74.27 %); however, only 66.83 % of women obtained PNC. The most frequent reasons for not obtaining PNC were lack of time (30.47 %), long distance to a provider (19.25 %), lack of guardians for children care (16.07 %), and lack of service (8.60 %). Based on the multivariate analysis, place of residence (AOR 2.68; 95 % CI 1.45–4.98), distance from a health institution (AOR 2.21; 95 % CI 1.39–3.51), antenatal care visit (AOR 2.60; 95 % CI 1.40–5.06), and having decision-making authority for utilization (AOR 1.86; 95 % CI 1.30–2.65) were factors found to be significantly associated with PNC utilization. Mothers in the study area had a high level of awareness and perception about the necessity of PNC. Urban women and those who displayed higher levels of autonomy were more likely to use postnatal health services.

## Background

The health of mothers is mostly regarded as an indicator the health of the society. Globally, more than half a million women die each year from complications of pregnancy and childbirth [1–3]. A large proportion of maternal and neonatal deaths occur during the first 48 h after delivery. Thus, postnatal care (PNC) is important for both the mother and the child to treat complications arising from the delivery, as well as to provide the mother with important information [4]. Every year, four million infants die within their first month of life, representing nearly 40 % of all deaths of children under age 5 year old. Almost all newborn deaths are in developing countries, with the highest number in South Asia and the highest rates in sub-Saharan Africa [5]. PNC coverage is low in Ethiopia; only 5 % of mothers received PNC within the critical first 2 days after delivery [4]. Nationwide, 34.3 % of mothers in Ethiopia receive PNC within the first 6 weeks after delivery. In the Amhara region (in which Gondar is found), the number of women attending PNC jumps to 44.5 % [6]. It is an incontrovertible fact that PNC services help to safeguard women from complications following delivery and provide important opportunities to assess the infant’s development. Moreover, PNC services help to offer newborn care (e.g. Counseling on breast feeding and Preventing Mother -to- Child Transmission) and other services like immunization and family planning which are crucial for both the mother and the infant [7–9].

In the course of a lifetime, an individual encounters the greatest risk of mortality during birth and the first 28 days of life (the neonatal period). The risk of maternal mortality and morbidity is also high at birth and in the immediate post-natal period. Each year, nearly 4 million newborns die during the neonatal period throughout the world. Three quarters of these deaths take place within 1 week of birth, 1–2 million die during the first day following birth, and most of these deaths occur at home [5]. The developing world has the highest prevalence of maternal and infant morbidity and mortality. A total of 99 % of all maternal deaths occur in developing countries, where 85 % of the world’s population lives [10]. This is especially true in sub-Saharan African countries. Over 13,000 mothers, newborns, and children die every day in sub-Saharan Africa [11]. Ethiopia, one of the countries in sub-Saharan Africa, is among the six countries that contribute about 50 % of the maternal deaths worldwide [12]. In 2005 in Ethiopia, the maternal mortality rate was found to be 673/100,000. This rate has declined to 470 in 2008 as reported in 2010/11 [4, 13, 14].

The poor quality of post-natal care in Ethiopia is a result of weak health infrastructure, poorly trained health professionals, and inadequate supplies of drugs and equipment. This, coupled with patients’ poverty, results in low utilization of services. According to the 2005 Ethiopia Demographic and Health Survey, an estimated 95 % of mothers nationwide did not receive PNC in the critical first 2 days after delivery [4]. Research indicates that maternal health care service utilization is affected by several factors including awareness of the health services, accessibility, socio-cultural beliefs, practices, individual attitudes, and health-care-seeking behaviors. However, the determinants of utilization of maternal health care services are not the same across different cultures and socioeconomic statuses within a society. There is little information about mothers’ knowledge, perception, and utilization of PNC and factors that influence the use of PNC in the Gondar Zuria District of Ethiopia. This study aimed to elucidate the various factors influencing the use of these services within the district.

## Methods

### Study Design and Study Setting

The study was cross-sectional and community-based, using both quantitative and qualitative methods of data collection and analysis. The study was conducted from April to August 2011 in the Gondar Zuria District, which is among twenty-four districts in the North Gondar Administrative Zone in the Amhara Regional State, is located 700 km from Addis Ababa, Ethiopia. At the time of the study, the population of the district was 204,698, of which 101,009 were female. There are 3 urban and 35 rural wards, five health centers, and two heath posts working to maintain the health status of people in the district.

### Sampling Procedure

The study population consisted of mothers from 15 to 49 years who gave birth in the last year in the selected wards and were residents of the district for at least 6 months. Multistage sampling technique was used to select study participants. The district was stratified into urban and rural wards, and then one urban and 12 rural wards were chosen by simple random sampling technique. In the study area, 12,282 women were estimated to be eligible (women who gave birth within the last year). The total sample size 836 was distributed proportionally to each strata: 131 urban and 705 rural households sampled were selected by systematic random sampling technique. If there was more than one mother within the same household, a lottery method was used to select the mother to be included.

Purposive sampling was used to select participants for focus group discussion (FGDs). Three FGDs which comprise a total of 6–8 individuals were conducted with mothers, health extension workers (HEWs), and community health workers (CHWs). A total of 16 mothers, three HEWs, and three CHWs participated in the FGDs.

### Data Collection Instrument

The questionnaire was developed through review of related Ethiopian and international literatures. The questionnaire was prepared in English then translated into Amharic which is the local language of the area and back to English in order to ensure its consistency. The questionnaires consisted of information on socio-demographic characteristics, knowledge and perception of mothers towards PNC, and utilization of PNC services. Pre-testing of the questionnaire was done in other, unselected wards of the district and modifications were made based on the outcome of the pre-test. The data were collected by interview using a structured questionnaire. For the qualitative data, guiding questions for the FDGs were developed in English and converted to Amharic and then checked for validity. Camera and tape recorders were used in the discussion to record every discussion on the topics. Data were collected by 13 diploma nursing students after a 2 days training. Three supervisors and the principal investigators closely followed the day to day data collection process and ensured completeness and consistency of the collected questionnaires. Each questionnaire was checked for completeness and consistency by supervisors and principal investigators and incompletely filled questionnaires were discarded.

### Data Processing and Analysis

The data were entered into EPI info version 3.5.1 and exported to SPSS version 16.0 for analysis. Descriptive statistics such as frequencies and percents were computed to describe the study population in relation to relevant variables. Bivariate and multivariate logistic regression analyses were employed. Those factors that were significant at the 20 % level in the Bivariate logistic regression analysis were considered for the multivariate logistic regression analysis. Odds ratios with 95 % Confidence Interval (CI) and *p* value of <0.05 were computed to assess the presence and degree of statistical association between dependent and independent variables. The qualitative data responses were coded, categorized, and then organized by content with thematic analysis.

### Ethical Considerations

Ethical clearance for the study was obtained from the Institutional Review Board of University of Gondar. Official letters were written from Institute of Public Health to North Gondar Health Office, to the District Health Office, and to each ward to get permission. Written and verbal consent was obtained from each participant after explaining the purpose and nature of the research. Participation in the study was on a voluntary basis and participants were informed their right to quit/refuse their participation at any stage of the study if they do not want to participate. Moreover, confidentiality of the information was assured by using an anonymous questionnaire.

## Result of the Study

### Socio-Demographic Characteristics of the Respondents

From the total 836 mothers 820 (98.09 %) completely filed and returned the questionnaire. The majority of mothers surveyed (84.88 %) were from rural areas, with the remainder (15.12 %) living in cities and towns. The mean age of study participants was 28.58 years, with a standard deviation of ±7.71 years. A large proportion of participants (47.32 %) travel a distance of one to 2 h on foot and 19.88 % require more than 2 h, whereas 32.80 % of participants travel <1 h to nearby health centers (Table 1).

**Table 1:** Socio-demographic and economic characteristics of respondents, North Gondar, Ethiopia, 2011 (n = 820)

Characteristic	Frequency	Percent (%)
Age (years)		
15–19	109	13.29
20–24	178	21.71
25–29	173	21.10
30–34	181	22.07
>35	179	21.83
Marital status		
Single	34	4.15
Married	706	86.10
Separated due to work	27	3.29
Divorced	33	4.02
Widowed	20	2.44
Educational level		
Illiterate	677	82.56
Primary	101	12.32
Secondary	33	4.02
Diploma and above	9	1.10
Husbands’ educational level of (n = 753)		
Illiterate	608	80.74
Primary	87	11.55
Secondary	37	4.91
Diploma and above	31	4.12
Mothers’ occupation		
Housewife	633	77.20
Farmer	81	9.88
Private employ	61	7.44
Government employ	10	1.22
Daily labor worker	33	4.02
Others	2	0.24
Husbands’ occupation (n = 753)		
Farmer	620	82.34
Private employ	68	9.03
Government employ	38	5.05
Daily labor worker	25	3.32
Others	2	0.27
Residence of participants		
Urban	124	15.12
Rural	696	84.88
Distance from health institution (h)		
<1	269	32.80
1–2	388	47.32
>2	163	19.88

### Awareness of Post Natal Care Service

Six hundred ninety-two (84.39 %) of mothers were aware that they should receive PNC services after delivery. Those women who were aware of the need for PNC cited the following reasons for attending a clinic in the post-natal period: 97.69 % of women mentioned the need to receive vaccinations; 42.49 % to be counseled on family planning; 37.57 % to prevent and treat delivery related problems; 22.98 % to receive nutritional advice; 7.08 % to discuss breastfeeding; 1.16 % to receive advice on danger signs of pregnancy.

Among mothers who were aware that they should receive PNC services after delivery 85.84 % were given information about PNC from HEWs, 17.77 % from nurses, 8.24 % from family, and 1.16 % from doctors.

Maternal age, marital status, place of residence, previous visit by community health agents/HEWs, and having follow up for antenatal care had association with awareness of mothers about PNC service in both bivariate and multivariate logistic regression analysis (*p* value <0.05) (Table 2).

**Table 2:** Association of factors with awareness of mothers about postnatal care service, North Gondar, Ethiopia, 2011 (n = 820)

Variable	Awareness of PNC		Crude OR (95 % CI)	Adjusted OR (95 % CI)
	Yes	No		
Age of participant (years)				
15–19	77	32	1.00	
20–24	151	27	2.32 (1.30–4.16)	2.56 (1.15–5.70)
25–29	150	23	2.71 (1.48–4.95)	3.16 (1.29–7.71)*
30–34	159	22	3.00 (1.64–5.51)	3.47 (1.31–9.18)*
>35	155	24	2.69 (1.48–4.87)*	2.93 (1.17–7.33)*
Marital status				
Single	17	17	1.00	
Married	616	90	6.84 (3.37–13.89)**	3.14 (1.17–8.41)*
Separated due to work	20	7	2.86 (0.96–8.52)	1.60 (0.37–6.81)
Divorced	25	8	3.13 (1.10–8.86)*	3.03 (0.77–11.98)
Widowed	14	6	2.33 (0.73–7.51)	2.41 (0.52–11.13)
Place of residence				
Rural	573	123	1.00	
Urban	119	5	5.11 (2.05–12.77)**	6.58 (2.09–20.75)*
Distance from health institution (h)				
<1	273	32	2.18 (1.29–3.66)	0.55 (0.27–1.12)
1–2	329	59	1.64 (1.03–2.59)*	0.73 (0.40–1.34)
>2	126	37	1.00	
Number of children born alive				
None	41	18	1.00	
1–5	372	65	2.51 (1.36–4.64)**	0.40 (0.16–1.01)
>5	279	45	2.72 (1.44–5.15)**	0.29 (0.09– 0.85)
ANC visit				
Not used	48	47	1.00	
Once	143	39	5.12 (3.07–8.55)**	2.82 (1.54–5.17)**
Twice	321	12	37.34 (18.82–74.08)**	26.50 (12.36–56.82)**
Three and more	180	10	25.03 (12.03–52.42)**	15.40 (6.76–35.08)**
Last 1 year visit health facility				
Yes	613	80	4.66 (3.04–8.55)**	1.44 (0.82––2.63)
No	79	48	1.00	
Visit by HEW				
Yes	18	77	4.08 (2.76–6.03)**	2.01 (1.19–3.39)**
No	505	51	1.00	
History of institutional delivery				
Yes	75	6	2.47 (1.05–5.81)	1.02 (0.39–2.68)
No	617	122	1.00	

1.00 = Referent category, * statistically associated *p* value <0.05, ** statistically associated *p* value <0.001

### Mothers’ Perception Towards Postnatal Care

The majority of mothers (74.27 %) stated that PNC is necessary for women and their children. Table 3 shows the factors that had an association with the positive perception to PNC.

**Table 3:** Association of factors with perception of mothers towards postnatal care service, North Gondar, Ethiopia, 2011 (n = 820)

Variable	Perceive positively		Crude OR (95 % CI)	Adjusted OR (95 % CI)
	Yes	No		
Age of participant(years)				
15–19	77	22	1.00	
20–24	151	27	2.54 (1.42–4.55)*	1.87 (0.98–3.55)
25–29	150	23	1.19 (0.71–2.04)	0.69 (0.38–1.27)
30–34	159	22	1.39 (0.82–2.38)	0.97 (0.54–1.76)
>35	155	24	0.79 (0.48–1.33)	0.59 (.33–1.05)
Place of residence				
Rural	487	209	1.00	
Urban	122	2	26.18 (6.41–11.86)**	11.22 (2.53–49.80)**
Distance from health Institution				
<1 h	240	29	2.60 (1.54–4.41)*	0.93 (0.50–1.71)
1–2 h	245	143	0.54 (0.36–0.82)	0.34 (0.22–0.58)
>2 h	124	39	1.00	
ANC visit				
Not used	59	56	1.00	
Once	156	26	5.70 (3.28–9.90)**	3.04 (1.61–5.74)*
Twice	249	84	2.81 (1.81–4.38)**	1.34 (0.74–2.41)
There and more	145	45	3.06 (1.86–5.02)**	1.26 (0.67–2.40)
Visit by community health agent/HEW				
Yes	451	105	2.88 (2.08–3.99)**	1.95 (1.33–2.86)**
No	158	106	1.00	
Awareness about PNC				
Yes	546	146	3.47 (2.61–5.71)**	3.55 (2.11–5.97)**
No	63	65	1.00	

1.00 = Referent category, * statistically associated *p* value <0.05, ** statistically associated *p* value <0.001

### Utilization of Post Natal Care

**Fig. 1:**
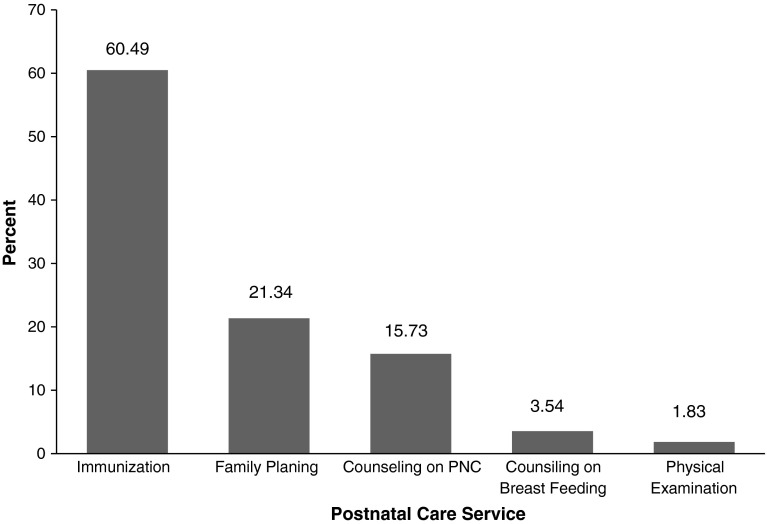
Utilization of postnatal care by mothers who give birth in the last 1 year, North Gondar, Ethiopia, 2011

Among mothers who give birth in the last 1 year, 548 (66.83 %) of them attended postnatal services. Above half 496 (60.49 %) of the mothers attended for immunization of their babies, 175 (21.34 %) for family planning, 129 (15.73 %) for counseling on PNC, 29 (3.54 %) for counseling on breastfeeding, and 15 (1.83 %) for physical examination (Fig. 1).

**Fig. 2:**
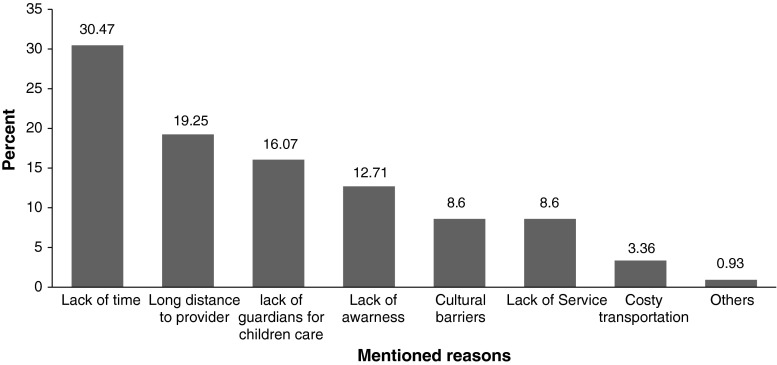
Mothers’ reasons for not attending postnatal care service, North Gondar, Ethiopia, 2011

Two hundred seventy-two (33.17 %) mothers who didn’t use PNC service provide different reasons for not attending PNC services. As depicted in (Fig. 2) majority of the mothers do not attend because of lack of time and the long distance required to travel in order to receive services.

Among mothers who utilize the service, 67.70 % utilized once, 27.92 % twice and 4.38 % three and or more times within 6 weeks after delivery. Half of (52.19 %) mothers utilize services from HEWs and community health agents in outreach service, 46.90 % of mothers from health institutions, and the other 0.91 % of mothers receive PNC from trained birth attendants.

In a multivariate logistic regression analysis, the following factors were associated with the utilization of PNC: place of residence, distance from health institution, history of ANC, contact with community health agents within the last year, awareness about the need for PNC, history of institutional delivery, and the ability to make decisions regarding healthcare (Table 4).

**Table 4:** Association of factors with post natal care utilization, North Gondar, Ethiopia, 2011 (n = 820)

Variable	PNC utilization		COR (95 % CI)	AOR (95 % CI)
	Yes	No		
Age of participant				
15–19	70	39	1.00	
20–24	106	72	0.82 (0.50–1.34)	0.50 (0.26–0.99)
25–29	116	57	1.13 (0.69–1.87)	0.55 (0.23–1.12)
30–34	116	65	0.99 (0.61–1.63)	0.51 (0.24–1.09)
>35	140	39	2.00 (1.18–3.39)*	1.03 (0.48–2.21)
Marital status				
Single	17	17	1.00	
Married	492	214	2.30 (1.15–4.59)*	1.50 (0.62–3.63)
Separated due to work	13	14	0.93 (0.34–2.55)	0.79 (0.24–2.58)
Divorced	15	18	0.83 (0.32–2.18)	1.10 (0.35–3.45)
Windowed	11	9	1.22 (0.40–3.70)	1.22 (0.33–4.48)
Place of residence				
Rural	449	247	1.00	
Urban	99	25	2.18 (1.37–3.47)**	2.68 (1.45–4.98)**
Distance from health institution (h)				
<1	183	32	2.10 (1.41–3.14)**	0.95 (0.56–1.62)
1–2	283	105	2.66 (1.82–3.89)**	2.21 (1.39–3.51)**
>2	82	81	1.00	
Number of children born alive				
None	30	29	1.00	
1–5	294	143	1.99 (1.15–3.44)*	2.11 (0.97–4.59)
>5	224	100	2.17 (1.23–3.80)*	1.83 (0.77–4.35)
Know about maternal health service				
Yes	279	97	1.87 (1.39–2.52)**	1.38 (0.97–1.96)
No	269	175	1.00	
ANC visit				
Not used	41	74	1.00	
Once	106	76	2.52 (1.55–4.08)**	1.36 (0.76–2.45)
Twice	253	80	5.71 (3.61–9.01)**	2.36 (1.31–4.23)*
There and more	148	42	6.36 (3.81–10.62)**	2.60 (1.40–5.06)*
Know about PNC service				
Yes	497	195	3.85 (2.60–5.69)**	1.72 (1.03–2.86)**
No	51	77	1.00	
Visit by community health agent/HEW				
Yes	410	146	2.56 (1.89–3.48)**	2.66 (1.39–5.06)**
No	138	126	1.00	
History of institutional delivery				
Yes	77	4	10.95 (3.97–30.26)**	8.09 (2.78–23.53)**
No	471	268		
Perception towards PNC				
Positive	420	189	1.44 (1.04–1.99)*	1.10 (0.72–1.68)
Negative	128	83		
Ability to make decisions for utilization				
Yes	196	152	2.28 (1.69–3.06)**	1.86 (1.31–2.65)**
No	352	120	1.00	

1.00 = Referent category, * statistically associated *p* value <0.05, ** statistically associated *p* value <0.001

### Results of the Focus Group Discussion

Focus group discussions were conducted in the Chira Mantebro, Des Denzez, and Degoma wards in order to triangulate the quantitative data. The qualitative data responses of the FDGs were grouped into three themes: awareness about PNC, perception towards the care, and factors associated with the utilization of PNC.

### Awareness of Post Natal Care Service

The majority of mothers demonstrated an awareness of immunization, family planning, and counseling on nutrition. Mothers reported that HEWs and community health agents informed them of the existence of PNC services. However, those who knew about the services did not have adequate information on when post-natal clinics are offered, or for whom. Most mothers assumed that the services were only given for children and vaccination 45 days after birth.

‘…Community health workers inform us about the vaccination and child nutrition but we didn’t practice because there is no full service here, the health facility is too far from here, and we do not have financial power…’ (A 32 year-old mother, focused group discussion, Chira Mantebro ward).

Similarly health extension works reported the existence of awareness on PNC among mothers. A HEA said: *‘…we usually told mothers about PNC services including immunization, counseling about breastfeeding, nutrition, and family planning however most mothers do have better awareness about vaccination…*’

‘… We usually inform the mothers on PNC services, and they do have an awareness about the service but most of them need to be motivated every time to use the service…’ *(*A community health worker, focused group discussion).

### Mothers’ Perceptions Toward PNC

Most participants in the discussions had a positive perception toward PNC and they encourage others to use PNC.

*‘…* Health extension workers gave a drop to my child 15 days after I deliver at home and it helps to my child. I went to the health post before 40 days for vaccination… it prevents my child form different diseases hence I want to have this service for my child to be healthy’ (A 25 year-old mother, focused group discussion, Des Denzaz Ward).

In harmony with the mother, a HEA stated *‘…most mothers in this ward posses positive feelings towards PNC services yet remoteness of the area prevent some mothers not to have the service…’’.*

*‘… Some mothers need to use PNC and they are happy to go to the health post for the service…’* (A community health worker)

### Factors Associated with the Utilization of Postnatal Care

Distance from health organization was a major problem, especially in remote, rural wards, some participants complain that they needed to walk on foot for up to 2 h to reach the nearest health center.

‘… A community health worker educate us about vaccination and how to care for our children but we fail to do because health post is too far and our husbands mostly did not allow us…’ (A 24 year-old mother, focused group discussion, Degoma Ward).

Congruent evidence was also gathered from a health extension workers: ‘… *there are village very far from here with no health extension workers so for such areas voluntary members of a society gave training and some services to mothers, but when we go for vaccination every month it is difficult to say they are getting the care …’*

Most women complained limited availability of health services (equipment and drugs): mainly in remote areas vaccines are less available. *‘… When my child gets sick there were no drug at the health post and full service even if they informed us about the care…’* (A 20 year-old mother, focused group discussion, Des Degoma Ward). ‘… *Only they vaccinate our children in our ward, there was no satisfactory service here and there was no drug …’ (*A 26 year-old mother, focused group discussion, Des Denzaz Ward).

Another constraint mentioned by mothers was absent or frequent travel of HEAs out of the ward. *‘… I gave birth before three months but when I went there after a first month for vaccination the health worker were not available…’* (A 32 year-old mother, focused group discussion, Chira Mantebro Ward).

Most mothers are responsible for house work such as baby care, preparation of food, and farming in rainy season which will delay their receiving of PNC services.

*‘… Community health worker told us about vaccination and counseled about nutrition but we have a work load to practice it…’* (A 22 year-old mother, focused group discussion, Des Denzaz Ward).

Some participants did not feel the need for PNC services unless their children and they were sick after delivery. ‘…*Since I did not get sick I did not go to the health post and never used family planning…’* (A 27 year-old mother, focused group discussion, Degoma Ward).

## Discussion

This community based study assessed knowledge, perception, and utilization of postpartum health care among women who give birth in the last year in Gondar Zuria District. The result showed that among the 820 postpartum women, 548 (66.83 %) obtained PNC during the 6 weeks following delivery. This is high compared with studies done in four other regions of Ethiopia (Amhara, Oromiya, Southern Nations, Nationalities and People’s, and Tigray) (10 %) [15], Sidama zone (37.2 %) [16], Uganda (58 %) [7], Nepal (34 %) [17], and Palestine (36.6 %) [18]. The difference may be attributed to time, place, and social context variation between the present study and previous studies.

In Ethiopia PNC coverage was 34.3 % and, in the case of Amhara region 44.5 %, which is lower than from the present finding [6]. The variation could be due to socio-demographic characteristics, methodology difference, implementation of the service, and accessibility of health organizations.

The government of Ethiopia endorsed a strategy which is aimed at strengthening the health system to provide quality care, particularly skilled attendance at birth and emergency obstetrical care through a functional referral system that includes zonal hospitals and four health centers that refer to it as well as health posts [19]. Through the Health Extension Program, the government plans to extend primary healthcare to the rural poor through deployment of about 30,000 government-salaried HEAs, two per kebele. Kebele Councils with Woreda Councils recruit young, locally resident women who have completed grade 10 and speak the local language to become Health Extension Workers (HEWs). Two HEWs are posted at a health post for a population of approximately 5,000; they are to spend 75 % of their time in outreach activities, teaching by example through three approaches: model families (40–60 families who are early adopters of desirable health practices), community organizations (e.g., Idir, Ekub, Mahber), and health post and outreach service delivery [20].

The majority (84.39 %) of mothers were aware that they were supposed to receive PNC services after delivery which is consistent with the studies done in Uganda (70.3 %) [7] and in Ethiopia [21]. Findings from the FGDs also showed that mothers had awareness and usually utilize the service 40 days after delivery. This high rate of awareness could be due to the range of government and non-government programs involved in the distribution of health information to the women such as the Integrated Family Health Program (IFPH) trained CHWs/agents for motivation of mothers to use maternal health services.

Postpartum maternal health care service awareness was associated with a previous visit by community health agents. Women who had antenatal follow up were 1.72 times more likely to be aware than women who did not have antenatal follow up. Similar studies in India show that mothers who attend antenatal care (64.4 %) were more aware than women who did not have (33.3 %) antenatal care [22]. It’s also similar to studies conducted in Indonesia, Nepal and Uganda [7, 17, 23]. The possible explanation might be that mothers receive health education and counseling during community and antenatal visits.

The majority of mothers (74.27 %) perceived that PNC is helpful to mothers, and children’s health, but only 66.83 % of mothers utilize PNC services. Those mothers who had awareness about PNC, who were previously visited by community health agents, and who had antenatal follow-up have most likely considered the care as important for maintaining their health and for their child’s health.

The most frequently cited reasons mentioned by the FGD participants for not utilizing PNC were believing that the treatments were not important unless mothers feel sick, negative experiences of women with the care, and considering the service accessible only for the child. Similar studies in Sweden show that women who had negative experiences were avoid PNC [24] other studies in Indonesia and the West Bank show that women in those areas have similar reasons to our study population for neglecting to utilize PNC [18, 23].

Place of residence, distance from a health organization, antenatal follow up, previous visit by community agents, and the ability to make a decision had effect on utilization of PNC service in this study. The participants in FGDs also raised these issues and stated that they affected the utilization of the service. Other studies on postpartum mothers revealed matching factors that have consequences for PNC utilization [17, 18, 22, 25–27].

In Bangladesh, urban mothers were more likely to receive PNC than rural mothers from both health professionals and non-health professionals [26]. Similarly, in this study, urban mothers were 2.68 times more likely to receive PNC than rural mothers (AOR 2. 68; 95 % CI 1.45–4.98). This might be due to the fact that urban women may have information from different sources on PNC or because of the availability of a good number of health institutions in urban areas.

Distance from the health institution remains a major problem as shown in previous literature; utilization of health services is strongly associated with access to health services [21, 23, 27–29]. In this study, one to 2 h travel to the nearby health centers resulted in women being 2.21 times more likely utilize the service than those who travel above 2 h. Comparable evidence was also gathered from FDG participants. This may be inferred to the cost of travel in terms of time, money, or energy. Mothers (71.91 %) who had antenatal care and mothers (73.74 %) who were previously visited by community health agents were more effective in utilizing PNC than those who had never been in any contact with the health system. This is in line with studies in Syria, Indonesia, Nepal, and Uganda [9, 18, 23, 30]. This could be due to the awareness of the mothers on possible postnatal complications as a result of previous contact with healthcare workers.

The ability to make a decision had a significant association with utilization of postpartum care. Mothers who can make a decision were more likely to use the service than those who cannot make a decision (AOR 1. 86; 95 % CI 1.31–2.65). Studies indicate autonomy of decision making as a factor in the utilization of maternal health services [9, 29, 31]. The possible reason for mothers not to make a decision might be the community belief about the hierarchy of authority in the household and economic dependency of mothers on husbands. A contradictory result with the current research was identified in a study in Ethiopia [32]. This may be attributed to the prevalence of paternalism in the study area.

Many findings in the literature indicate a significant association of PNC utilization with the occupation of women, education of women, and the husbands’ occupation [9, 17, 18, 33]. On the other hand, this study revealed no significant association with these variables. The majority of the respondents were illiterate, housewives and farmers which may be contributing to the lack of association in the study area.

## Conclusion

The majority of the mothers had an awareness of PNC services but they did not know when they should seek those services. From the result it can be concluded that mothers’ awareness about PNC service is more focused on the immunization component than others. Most mothers have a positive perception toward PNC services; however, mothers in a rural area possess a negative perception. PNC utilization was high but utilization of the most crucial elements was very low and large segments of mothers utilize only immunization services. Place of residence, distance from health institution, antenatal follow-up, previous visit by community health agents, and the ability to make decisions were significant factors that influence utilization of postnatal services.

## Abbreviations

ANC: Antenatal care
AOR: Adjusted odd ration
CHWs: Community health workers
CI: Confidence interval
FGD: Focus group discussion
HEW: Health extension worker
IFPH: Integrated family health program
OR: Odd ratio
PNC: Postnatal care
SPSS: Statistical package for the social sciences
